# Synthesis and crystal structure of (±)-Goniotamirenone C

**DOI:** 10.1107/S2056989020013298

**Published:** 2020-10-09

**Authors:** Pornphimol Meesakul, Christopher Richardson, Surat Laphookhieo, Stephen G. Pyne

**Affiliations:** a Center of Chemical Innovation for Sustainability (CIS) and School of Science, Mae Fah Luang University, Chiang Rai 57100, Thailand; b School of Chemistry and Molecular Bioscience, University of Wollongong, Wollongong, New South Wales 2522, Australia

**Keywords:** crystal structure, Goniotamirenone C, natural product, semi-synthesis, chloro­hydrin

## Abstract

The structure of the racemic version of the natural product Goniotamirenone C [racemic *anti*-6-(2-chloro-1-hy­droxy-2-phenyl­eth­yl)-2*H*-pyran-2-one, C_13_H_11_ClO_3_] at 150 K is reported. The compound crystallizes with monoclinic (*P*2_1_/*n*) symmetry and with *Z*′ = 2. One independent mol­ecule is ordered while the other independent mol­ecule exhibits an inter­esting whole-mol­ecule enanti­omeric disorder with occupancies of 0.846 (4) and 0.154 (4). The independent mol­ecules are hydrogen bonded with –OH⋯O=C linkages into chains that run parallel to the *a* axis. This structural analysis corrects our previous assignment as the *syn* isomer [Meesakul *et al.* (2020 ▸). *Phytochemistry*, **171**, 112248–112255].

## Abstract 2

Earlier we reported the isolation of Goniotamirenone C [6-(2-chloro-1-hy­droxy-2-phenyl­eth­yl)-2*H*-pyran-2-one] from the leaf extracts of *Goniothalamus tamirensis*. Its gross structure was elucidated using NMR spectroscopic techniques and, on the basis of ECD calculations, the absolute configuration of this natural product was assigned as the *syn* isomer, (7*S*,8S)-Goniotamirenone C. In this paper we correct the structure of the natural product to the *anti* isomer from the semi-synthesis and single-crystal X-ray structure determination of (±)-Goniotamirenone C, which displays identical NMR spectroscopic data to the natural product.

## Chemical context   

*Goniothalamus* is one of the largest genera belonging to the Annona­ceae family, which is distributed throughout tropical and subtropical areas. So far, over 160 species have been discovered globally (Saunders & Chalermglin, 2008 ▸) with 15 species found in Thailand (Soonthornchareonnon *et al.*, 1999 ▸). Many species of *Goniothalamus* have been used as folk medicines for the treatment of common illnesses and as a tonic. *Goniothalamus* is well known as a rich source of styryllactones, with over 100 compounds isolated and identified (Meesakul *et al.*, 2020 ▸; Jaidee *et al.*, 2019 ▸, Bihud *et al.*, 2019 ▸). However, chlorinated styryllactones are rarely reported in the Annona­ceae family. To the best of our knowledge, only two compounds, Parvistone A and Goniotamirenone C, have been isolated and identified from *Polyalthia parviflora* (Liou *et al.*, 2014 ▸) and *Goniothalamus tamirensis* (Meesakul *et al.*, 2020 ▸), respectively. Styryllactones show inter­esting pharmacological activities, such as cytotoxic activity against several tumor cell lines (Lan *et al.*, 2005 ▸; Tian *et al.*, 2006 ▸; Prawat *et al.*, 2012 ▸), anti­mycobacterial (Lekphrom *et al.*, 2009 ▸; Prawat *et al.*, 2012 ▸) and anti­plasmodial activities (Lekphrom *et al.*, 2009 ▸; Prawat *et al.*, 2012 ▸). As a part of our continuing study of the phytochemistry of plants in the Annona­ceae family, we report here the synthesis and crystal structure of (±)-Goniotamirenone C.
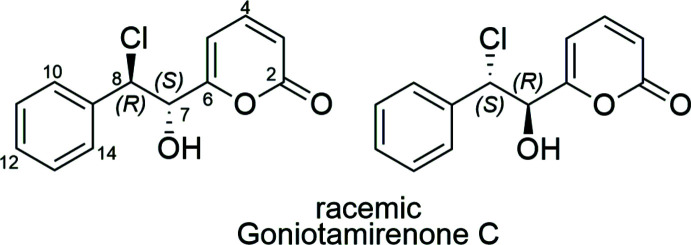


## Structural commentary   

**Figure 1:**
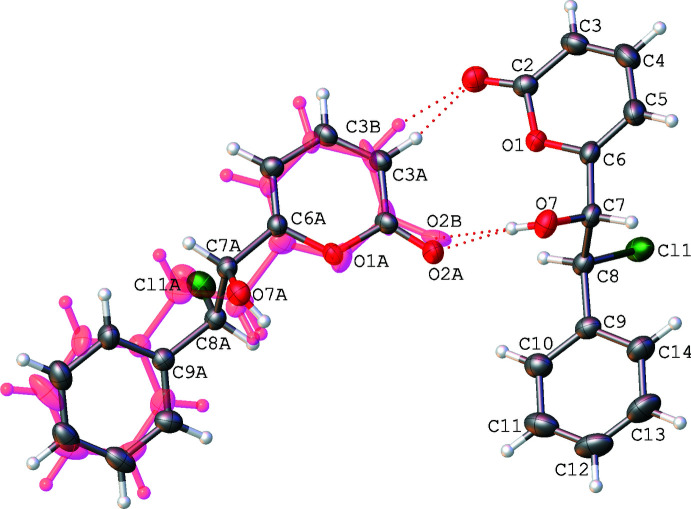
The contents of the asymmetric unit with complete atom labelling of the ordered mol­ecule and selected labelling of major and minor occupancy disordered mol­ecules, for clarity. The minor occupancy mol­ecule is shaded in pink. Displacement ellipsoids are plotted at the 50% probability level.

The title compound crystallizes in the space group *P*2_1_/*n* with *Z*′ = 2. The centrosymmetric space group confirms the compound crystallizes as a racemic mixture. One mol­ecule is ordered within the asymmetric unit and there is disorder of the other mol­ecular site with occupancies of 0.846 (4) and 0.154 (4) (Fig. 1 ▸). The mol­ecules have two stereogenic carbon centres and the ordered mol­ecule has the configuration (7*R*,8*S*), in the asymmetric unit selected. The major occupancy component on the disordered site is of configuration (7*S*,8*R*) and the configuration of the minor component is (7*R*,8*S*). Thus the minor component of the disorder has the same configuration as the ordered mol­ecule in the selected asymmetric unit. These assignments confirm the relative stereochemistry as *anti* and thus the structural assignment can be revised from our earlier work (Meesakul *et al.* 2020 ▸).

Each mol­ecule adopts a staggered conformation about the bond between the stereocentres with chlorine and hydroxyl groups anti­periplanar (Table 1 ▸). The main conformational difference between mol­ecules on the ordered site and the disordered site is the dihedral angle between the phenyl (C9*X*–C14*X*; where *X* takes no value for the ordered site and *A* and *B* for the disordered site) and pyran-2-one rings (O1*X*, C2*X*–C6*X*). This angle is only 5.88 (6)° on the ordered site and 28.22 (18)° and 27.7 (11)°, respectively, for the major and minor occupancy mol­ecules on the disordered site.

## Supra­molecular features   

**Figure 2:**
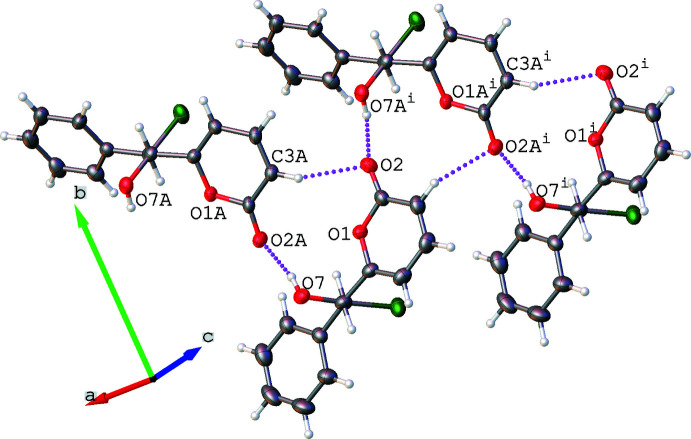
A perspective view, with hydrogen bonds shown as dotted magenta lines, of a part of one chain that propagates along the [100] direction. Symmetry code: (i) −1 + *x*, +*y*, +*z*. Displacement ellipsoids are plotted at the 50% probability level.

**Figure 3:**
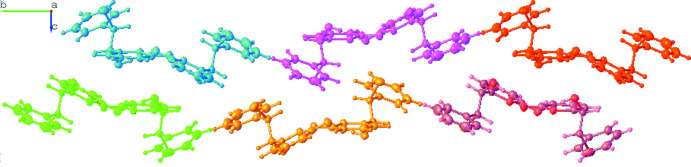
A view along the [100] direction of the stacking of the hydrogen-bonded chains. Chains are coloured differently for clarity.

The mol­ecules in the asymmetric unit are linked by hydrogen bonds between the hydroxyl groups as hydrogen-bond donors and the carbonyl groups of the lactones as hydrogen-bond acceptors. The hydrogen-bond metrics are presented in Table 2 ▸ and fall within standard values. These inter­actions link the mol­ecules into chains running parallel to the [100] direction (Fig. 2 ▸). For clarity, the inter­actions between the ordered mol­ecule and the major component of the disorder are shown. These O—H⋯O inter­actions are supported by C–H⋯O=C inter­actions within the chain. The chains stack, seemingly rather awkwardly, in the [001] direction (Fig. 3 ▸), presenting an inter­esting C5—H5⋯Cl (2.70 Å) inter-chain contact.

## Synthesis and crystallization   

**Figure 4:**
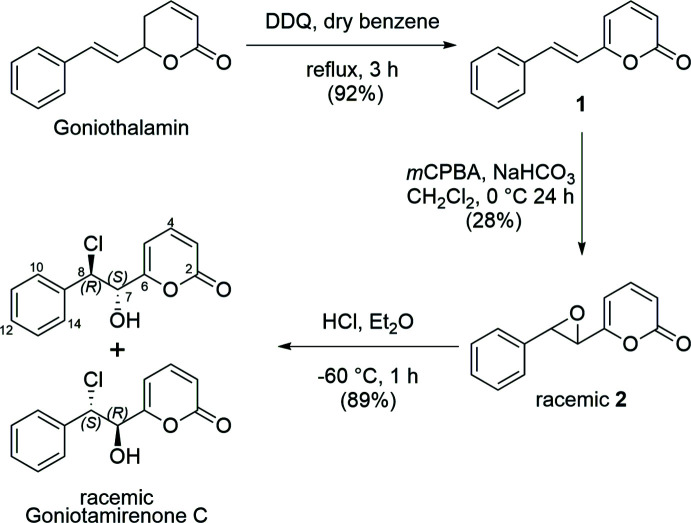
Synthesis of (±)-Goniotamirenone C.

The synthetic sequence starts by de­hydrogenating the natural product Goniothalamin by reaction with 2,3-di­chloro-5,6-di­cyano-1,4-benzo­quinone (DDQ) in refluxing benzene solution to give (*E*)-6-styryl­pyran-2-one (**1**) in 92% yield (Fig. 4 ▸). The central alkene unit in **1** was epoxidized selectively under basic conditions using *meta*-chloro­perbenzoic acid (*m*CPBA) in di­chloro­methane solution at 273 K to give racemic 6-[3-phenyl-2-oxiran­yl]-2*H*-pyran-2-one (**2**), albeit in 28% yield. Compound **2** was ring-opened at 213 K using HCl in anhydrous diethyl ether solution, furnishing the desired compound as a colourless solid. Crystals suitable for analysis by single crystal X-ray diffraction grew from slow evaporation of a 1:4 di­chloro­methane:methanol solution.

**(** ***E*** **)-6-Styryl­pyran-2-one (1)**

2,3-Di­chloro-5,6-di­cyano-1,4-benzo­quinone (DDQ; 52.8 mg, 0.52 mmol) was added to a solution of Goniothalamin (44.0 mg, 0.44 mmol), isolated as described previously (Meesakul *et al.*, 2020 ▸), in anhydrous benzene (5 mL) and the solution heated at reflux for 3 h. The cooled crude mixture was filtered through Celite and concentrated under reduced pressure. Purification of the residue by column chromatography (EtOAc/*n*-hexane, 1:5) yielded **1** (43.8 mg, 92%) as a yellow solid after evaporation of the solvent.

M.p. 387–388 K [lit. (Thibonnet *et al.*, 2002 ▸) 388–389 K]; ^1^H NMR (CDCl_3_, 500 MHz) *δ*_H_ 6.21 (1H, *d*, *J* = 9.0 Hz, H-3), 6.14 (1H, *d*, *J* = 6.7 Hz, H-5), 6.62 (1H, *d*, *J* = 16.0 Hz, H-8), 7.39–7.29 (4H, *m*, H-4, H-11, H-12, H-13), 7.53–7.44 (3H, *m*, H-7, H-10, H-14); ^13^C NMR (CDCl_3_, 125 MHz) *δ*_C_ 161.8 (C-2), 114.3 (C-3), 143.7 (C-4), 105.0 (C-5), 159.7 (C-6), 135.4 (C-7), 118.8 (C-8), 135.3 (C-9), 127.4 (C-10, C-14), 128.9 (C-11, C-13), 129.5 (C-12).

**(**±**)-6-[3-Phenyl-2-oxiran­yl]-2** ***H*** **-pyran-2-one (2)**

NaHCO_3_ (84 mg, 1.0 mmol) followed by *m*CPBA (64 mg, 0.4 mmol) were added to a stirred solution of **1** (19.8 mg, 0.1 mmol) in CH_2_Cl_2_ (2 mL) at 273.15 K and then stirred at room temperature for 24 h. The mixture was quenched by the addition of saturated aqueous NaHCO_3_ (3 mL) and water (3 mL) and extracted with EtOAc (8 mL). Purification by column chromatography (EtOAc/*n*-hexane, 1:3) yielded **2** (5.6 mg, 28%) as a white solid after evaporation of the solvent,

M.p. 393–396 K; ^1^H NMR (CDCl_3_, 500 MHz) *δ*_H_ 6.26–6.32 (2H, *m*, H-3, H-5), 3.64 (1H, *d*, *J* = 1.8 Hz, H-7), 4.18 (1H, *d*, *J* = 1.8 Hz, H-8), 7.30–7.38 (6H, *m*, H-4, H-10 to H-14); ^13^C NMR (CDCl_3_, 125 MHz) *δ*_C_ 161.2 (C-2), 115.8 (C-3), 143.0 (C-4), 103.4 (C-5), 159.8 (C-6), 58.3 (C-7), 60.8 (C-8), 135.2 (C-9), 125.7 (C-10, C-14), 128.8 (C-11, C-13), 129.0 (C-12).

**(**±**)-Goniotamireone C**

2 *M* HCl in Et_2_O (0.023 mL, 0.046 mmol) was added to a solution of **2** (12.0 mg, 0.056 mmol) in CHCl_3_ (1 mL) and stirred at 213 K for 2h. The reaction was quenched by the addition of saturated aqueous NaHCO_3_ (3 mL) then extracted using EtOAc and purified by column chromatography (EtOAc/*n*-hexane, 2:5) to yield (±)-Goniotamireone C (10.7 mg, 89%) as a white solid. The NMR spectroscopic data were identical to that of natural Goniotamirenone C (Meesakul *et al.*, 2020 ▸).

M.p. 394–396 K; ^1^H NMR (CDCl_3_, 500 MHz) *δ*_H_ 6.23 (1H, *d*, *J* = 9.4 Hz, H-3), 7.24 (1H, *dd*, *J* = 9.4,6.2 Hz. H-4), 6.17 (1H, *d*, *J* = 6.4 Hz, H-5), 4.81 (1H, *d*, *J* = 6.2 Hz, H-7), 5.28 (1H, *d*, *J* = 6.2 Hz, H-8), 7.39–7.38 (2H, *m*, H-10, H-14), 7.34–7.35 (3H, *m*, H-11, H-12, H-13); ^13^C NMR (CDCl_3_, 125 MHz) *δ*_C_ 161.4 (C-2), 115.1 (C-3), 143.1 (C-4), 104.1 (C-5), 161.3 (C-6), 74.9 (C-7), 62.6 (C-8), 136.0 (C-9), 128.2 (C-10, C-14), 128.6 (C-11, C-13), 129.1 (C-12).

## Refinement   

Crystal data, data collection and structure refinement details are summarized in Table 3 ▸. The disorder was modelled by reference to a free variable and the refined disorder occupancies are 0.846 (4) and 0.154 (4). The bond distances and 1,3-non-bonded distances in the pyran-2-one and chloro­hydrin parts of the minor disordered component were restrained to be the same as the corresponding distances in the ordered independent mol­ecule, subject to s.u. values of 0.02 and 0.04 Å, respectively, while the phenyl group of this mol­ecule was fitted as a regular hexa­gon and refined as free rotating group. Enhanced rigid bond restraints were applied to the pyran-2-one ring of the minor component. The anisotropic displacement parameters for the Cl atoms in the disordered mol­ecules were constrained to be identical. H atoms bonded to C atoms were located in difference maps for the ordered independent mol­ecule and the major component on the disordered site. All C-bound H atoms were placed in geometrically idealized positions with bond lengths of 0.95 Å (aromatic C-H) and 1.00 Å (aliphatic C—H), and refined using riding models with *U*_iso_(H) = 1.2*U*_eq_(C). H atoms attached to O were refined using riding models with *U*_iso_(H) = 1.5*U*_eq_(O) and as freely rotating idealized tetra­hedral groups with bond lengths of 0.84 Å.

## Supplementary Material

Crystal structure: contains datablock(s) I. DOI: 10.1107/S2056989020013298/vm2241sup1.cif

Supporting information file. DOI: 10.1107/S2056989020013298/vm2241Isup2.cml

CCDC reference: 1842573

Additional supporting information:  crystallographic information; 3D view; checkCIF report

## Figures and Tables

**Table 1:** Selected torsion angles (°)

O7—C7—C8—Cl1	176.98 (10)	O7*B*—C7*B*—C8*B*—Cl1*B*	177.9 (11)
O7*A*—C7*A*—C8*A*—Cl1*A*	−179.6 (3)		

**Table 2:** Hydrogen-bond geometry (Å, °)

*D*—H⋯*A*	*D*—H	H⋯*A*	*D*⋯*A*	*D*—H⋯*A*
O7—H7*A*⋯O2*A*	0.84	1.95	2.775 (4)	169
O7—H7*A*⋯O2*B*	0.84	1.79	2.63 (3)	173
O7*A*—H7*AB*⋯O2^i^	0.84	2.01	2.835 (6)	170
O7*B*—H7*BA*⋯O2^i^	0.84	1.86	2.63 (3)	153
C3—H3⋯O2*A* ^ii^	0.95	2.33	3.220 (6)	155
C3—H3⋯O2*B* ^ii^	0.95	2.52	3.42 (4)	158
C3*A*—H3*A*⋯O2	0.95	2.36	3.236 (5)	153
C5—H5⋯Cl1^iii^	0.95	2.70	3.6042 (17)	159

Symmetry codes: (i) 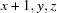; (ii) 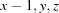; (iii) 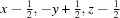.

**Table 3:** Experimental details

Crystal data	
Chemical formula	C_13_H_11_ClO_3_
*M*_r_	250.67
Crystal system, space group	Monoclinic, *P*2_1_/*n*
Temperature (K)	150
*a*, *b*, *c* (Å)	8.79348 (19), 27.8665 (5), 10.2288 (3)
β (°)	112.393 (3)
*V* (Å^3^)	2317.49 (10)
*Z*	8
Radiation type	Mo *K*α
μ (mm^−1^)	0.32
Crystal size (mm)	0.44 × 0.26 × 0.14

Data collection	
Diffractometer	Rigaku XtaLAB Mini II
Absorption correction	Multi-scan (*CrysAlis PRO*; Rigaku OD, 2018 ▸)
*T*_min_, *T*_max_	0.793, 1.000
No. of measured, independent and observed [*I* > 2σ(*I*)] reflections	53946, 5716, 4902
*R*_int_	0.034
(sin θ/λ)_max_ (Å^−1^)	0.667

Refinement	
*R*[*F* ^2^ > 2σ(*F* ^2^)], *wR*(*F* ^2^), *S*	0.040, 0.103, 1.08
No. of reflections	5716
No. of parameters	443
No. of restraints	64
H-atom treatment	H-atom parameters constrained
Δρ_max_, Δρ_min_ (e Å^−3^)	0.81, −0.31

Computer programs: *CrysAlis PRO* (Rigaku OD, 2018 ▸), *SHELXT* (Sheldrick, 2015*b* ▸), *SHELXL* (Sheldrick, 2015*a* ▸) and *OLEX2* (Dolomanov *et al.*, 2009 ▸).

## supplementary crystallographic information

### Crystal data

**Table:**

C_13_H_11_ClO_3_	*D*_x_ = 1.437 Mg m^−^^3^
*M**_r_* = 250.67	Melting point = 394–396 K
Monoclinic, *P*2_1_/*n*	Mo *K*α radiation, λ = 0.71073 Å
*a* = 8.79348 (19) Å	Cell parameters from 21920 reflections
*b* = 27.8665 (5) Å	θ = 2.3–30.4°
*c* = 10.2288 (3) Å	µ = 0.32 mm^−^^1^
β = 112.393 (3)°	*T* = 150 K
*V* = 2317.49 (10) Å^3^	Block, colourless
*Z* = 8	0.44 × 0.26 × 0.14 mm
*F*(000) = 1040	

### Data collection

**Table:**

Rigaku XtaLAB Mini II diffractometer	5716 independent reflections
Radiation source: fine-focus sealed X-ray tube, Rigaku (Mo) X-ray Source	4902 reflections with *I* > 2σ(*I*)
Graphite monochromator	*R*_int_ = 0.034
ω scans	θ_max_ = 28.3°, θ_min_ = 2.3°
Absorption correction: multi-scan (CrysAlisPro; Rigaku OD, 2018)	*h* = −11→11
*T*_min_ = 0.793, *T*_max_ = 1.000	*k* = −37→37
53946 measured reflections	*l* = −13→13

### Refinement

**Table:**

Refinement on *F*^2^	Primary atom site location: dual
Least-squares matrix: full	Hydrogen site location: mixed
*R*[*F*^2^ > 2σ(*F*^2^)] = 0.040	H-atom parameters constrained
*wR*(*F*^2^) = 0.103	*w* = 1/[σ^2^(*F*_o_^2^) + (0.0419*P*)^2^ + 1.0958*P*] where *P* = (*F*_o_^2^ + 2*F*_c_^2^)/3
*S* = 1.08	(Δ/σ)_max_ < 0.001
5716 reflections	Δρ_max_ = 0.81 e Å^−^^3^
443 parameters	Δρ_min_ = −0.31 e Å^−^^3^
64 restraints	

### Special details

**Table:**

Geometry. All esds (except the esd in the dihedral angle between two l.s. planes) are estimated using the full covariance matrix. The cell esds are taken into account individually in the estimation of esds in distances, angles and torsion angles; correlations between esds in cell parameters are only used when they are defined by crystal symmetry. An approximate (isotropic) treatment of cell esds is used for estimating esds involving l.s. planes.
Refinement. Approximately 15% of the opposite enantiomer crystallises about the same position as one of the two independent molecules in the asymmetric unit. This was modelled using PART instructions and by using the SAME command for the minor component to the appropriate ordered molecule and the RIGU restraint. The refinement settled well.

### Fractional atomic coordinates and isotropic or equivalent isotropic displacement parameters (Å^2^)

**Table:**

	*x*	*y*	*z*	*U*_iso_*/*U*_eq_	Occ. (<1)
Cl1	0.32805 (5)	0.27929 (2)	0.32248 (5)	0.03931 (12)	
O1	0.19539 (12)	0.38851 (4)	0.14334 (12)	0.0280 (2)	
C2	0.06986 (19)	0.41632 (6)	0.15322 (16)	0.0291 (3)	
O2	0.11024 (14)	0.45514 (4)	0.21042 (13)	0.0358 (3)	
C3	−0.0917 (2)	0.39555 (6)	0.09867 (19)	0.0371 (4)	
H3	−0.180630	0.412327	0.108707	0.045*	
C4	−0.1185 (2)	0.35297 (7)	0.0340 (2)	0.0410 (4)	
H4	−0.226415	0.339879	−0.002256	0.049*	
C5	0.0135 (2)	0.32706 (6)	0.01912 (19)	0.0375 (4)	
H5	−0.006149	0.297389	−0.030281	0.045*	
C6	0.16559 (19)	0.34516 (5)	0.07573 (16)	0.0286 (3)	
C7	0.32159 (19)	0.32161 (5)	0.08075 (16)	0.0285 (3)	
H7	0.292338	0.289992	0.031228	0.034*	
O7	0.40270 (15)	0.34945 (4)	0.01160 (12)	0.0344 (3)	
H7A	0.450794	0.372588	0.063544	0.052*	
C8	0.43932 (19)	0.31233 (5)	0.23401 (17)	0.0284 (3)	
H8	0.469 (2)	0.3433 (7)	0.2880 (19)	0.034*	
C9	0.5936 (2)	0.28604 (6)	0.24594 (17)	0.0310 (3)	
C10	0.7444 (2)	0.30839 (7)	0.31029 (19)	0.0377 (4)	
H10	0.749903	0.339773	0.348115	0.045*	
C11	0.8882 (2)	0.28497 (8)	0.3197 (2)	0.0489 (5)	
H11	0.991429	0.300479	0.363557	0.059*	
C12	0.8810 (3)	0.23955 (8)	0.2659 (2)	0.0541 (5)	
H12	0.979428	0.223647	0.273004	0.065*	
C13	0.7311 (3)	0.21688 (8)	0.2013 (2)	0.0538 (5)	
H13	0.726550	0.185437	0.164088	0.065*	
C14	0.5870 (2)	0.24015 (6)	0.1907 (2)	0.0423 (4)	
H14	0.483896	0.224657	0.145734	0.051*	
Cl1A	0.93903 (13)	0.57893 (5)	0.49691 (10)	0.0377 (2)	0.846 (4)
O1A	0.7230 (4)	0.49234 (15)	0.2049 (4)	0.0260 (6)	0.846 (4)
C2A	0.5818 (5)	0.46527 (14)	0.1782 (7)	0.0268 (10)	0.846 (4)
O2A	0.5979 (6)	0.42183 (13)	0.1788 (7)	0.0329 (8)	0.846 (4)
C3A	0.4349 (5)	0.49166 (15)	0.1631 (8)	0.0289 (8)	0.846 (4)
H3A	0.336315	0.474783	0.150149	0.035*	0.846 (4)
C4A	0.4360 (5)	0.53973 (17)	0.1673 (8)	0.0292 (8)	0.846 (4)
H4A	0.338434	0.556466	0.158285	0.035*	0.846 (4)
C5A	0.5820 (4)	0.56627 (14)	0.1851 (4)	0.0279 (6)	0.846 (4)
H5A	0.581503	0.600358	0.184988	0.033*	0.846 (4)
C6A	0.7197 (4)	0.54175 (13)	0.2019 (5)	0.0255 (7)	0.846 (4)
C7A	0.8852 (2)	0.56233 (7)	0.2189 (2)	0.0252 (4)	0.846 (4)
H7AA	0.871051	0.597715	0.202525	0.030*	0.846 (4)
O7A	0.9428 (6)	0.5433 (2)	0.1172 (4)	0.0296 (8)	0.846 (4)
H7AB	0.980517	0.515579	0.141834	0.044*	0.846 (4)
C8A	1.0153 (2)	0.55472 (7)	0.3678 (2)	0.0260 (5)	0.846 (4)
H8A	1.031853	0.519406	0.384443	0.031*	0.846 (4)
C9A	1.1797 (3)	0.57712 (10)	0.3931 (2)	0.0263 (4)	0.846 (4)
C10A	1.1931 (3)	0.62302 (12)	0.3442 (3)	0.0329 (6)	0.846 (4)
H10A	1.096421	0.640320	0.290083	0.040*	0.846 (4)
C11A	1.3472 (5)	0.64377 (11)	0.3740 (4)	0.0398 (7)	0.846 (4)
H11A	1.355195	0.675081	0.340479	0.048*	0.846 (4)
C12A	1.4879 (5)	0.61874 (18)	0.4524 (6)	0.0413 (10)	0.846 (4)
H12A	1.592855	0.632815	0.472912	0.050*	0.846 (4)
C13A	1.4758 (6)	0.57314 (17)	0.5009 (5)	0.0409 (12)	0.846 (4)
H13A	1.572739	0.556021	0.555407	0.049*	0.846 (4)
C14A	1.3225 (6)	0.55226 (13)	0.4703 (4)	0.0339 (7)	0.846 (4)
H14A	1.315444	0.520686	0.502562	0.041*	0.846 (4)
Cl1B	0.9422 (9)	0.5930 (2)	0.4962 (7)	0.0377 (2)	0.154 (4)
O1B	0.722 (3)	0.4848 (8)	0.206 (4)	0.050 (7)	0.154 (4)
C2B	0.566 (3)	0.4672 (8)	0.186 (4)	0.038 (6)	0.154 (4)
O2B	0.561 (3)	0.4241 (9)	0.156 (4)	0.040 (6)	0.154 (4)
C3B	0.437 (3)	0.5015 (8)	0.151 (5)	0.039 (7)	0.154 (4)
H3B	0.326106	0.491106	0.113594	0.047*	0.154 (4)
C4B	0.471 (3)	0.5473 (9)	0.170 (4)	0.026 (4)	0.154 (4)
H4B	0.387210	0.569887	0.162666	0.031*	0.154 (4)
C5B	0.633 (2)	0.5624 (8)	0.202 (3)	0.034 (5)	0.154 (4)
H5B	0.654777	0.595892	0.207136	0.041*	0.154 (4)
C6B	0.7555 (18)	0.5324 (7)	0.226 (3)	0.033 (5)	0.154 (4)
C7B	0.9378 (11)	0.5427 (4)	0.2782 (11)	0.025 (2)	0.154 (4)
H7B	0.999780	0.516699	0.344603	0.030*	0.154 (4)
O7B	0.975 (3)	0.5408 (11)	0.1509 (18)	0.023 (3)	0.154 (4)
H7BA	1.035535	0.517152	0.155396	0.035*	0.154 (4)
C8B	0.9942 (12)	0.5899 (4)	0.3448 (11)	0.035 (3)	0.154 (4)
H8B	0.931756	0.615484	0.277003	0.042*	0.154 (4)
C9B	1.1750 (11)	0.5987 (6)	0.3843 (12)	0.0263 (4)	0.154 (4)
C10B	1.236 (2)	0.6409 (5)	0.3507 (11)	0.034 (3)	0.154 (4)
H10B	1.162789	0.665177	0.297904	0.041*	0.154 (4)
C11B	1.405 (2)	0.6475 (6)	0.394 (2)	0.058 (7)	0.154 (4)
H11B	1.446986	0.676333	0.371466	0.069*	0.154 (4)
C12B	1.5126 (12)	0.6119 (9)	0.472 (3)	0.044 (8)	0.154 (4)
H12B	1.628029	0.616455	0.501574	0.052*	0.154 (4)
C13B	1.4514 (18)	0.5697 (8)	0.505 (3)	0.053 (12)	0.154 (4)
H13B	1.524873	0.545420	0.558122	0.064*	0.154 (4)
C14B	1.283 (2)	0.5631 (5)	0.4616 (19)	0.042 (7)	0.154 (4)
H14B	1.240675	0.534262	0.484561	0.050*	0.154 (4)

### Atomic displacement parameters (Å^2^)

**Table:**

	*U*^11^	*U*^22^	*U*^33^	*U*^12^	*U*^13^	*U*^23^
Cl1	0.0370 (2)	0.0389 (2)	0.0482 (3)	0.00724 (16)	0.02318 (19)	0.01609 (18)
O1	0.0251 (5)	0.0239 (5)	0.0350 (6)	−0.0001 (4)	0.0115 (4)	−0.0013 (4)
C2	0.0291 (7)	0.0319 (8)	0.0297 (8)	0.0037 (6)	0.0150 (6)	0.0033 (6)
O2	0.0362 (6)	0.0347 (6)	0.0414 (7)	0.0021 (5)	0.0201 (5)	−0.0052 (5)
C3	0.0261 (8)	0.0442 (9)	0.0423 (9)	0.0030 (7)	0.0144 (7)	0.0061 (7)
C4	0.0268 (8)	0.0450 (10)	0.0459 (10)	−0.0062 (7)	0.0078 (7)	0.0049 (8)
C5	0.0352 (9)	0.0316 (8)	0.0394 (9)	−0.0052 (7)	0.0071 (7)	−0.0013 (7)
C6	0.0319 (8)	0.0216 (7)	0.0299 (8)	−0.0006 (6)	0.0090 (6)	0.0019 (6)
C7	0.0329 (8)	0.0211 (6)	0.0328 (8)	−0.0007 (6)	0.0137 (6)	0.0001 (6)
O7	0.0452 (7)	0.0256 (5)	0.0382 (6)	−0.0013 (5)	0.0224 (5)	0.0012 (4)
C8	0.0312 (7)	0.0235 (7)	0.0341 (8)	0.0010 (6)	0.0163 (6)	0.0018 (6)
C9	0.0318 (8)	0.0316 (8)	0.0326 (8)	0.0059 (6)	0.0156 (6)	0.0053 (6)
C10	0.0330 (8)	0.0406 (9)	0.0417 (9)	0.0032 (7)	0.0167 (7)	0.0042 (7)
C11	0.0317 (9)	0.0644 (13)	0.0525 (12)	0.0074 (8)	0.0181 (8)	0.0119 (10)
C12	0.0471 (11)	0.0625 (13)	0.0638 (13)	0.0251 (10)	0.0335 (10)	0.0163 (11)
C13	0.0629 (13)	0.0418 (11)	0.0647 (14)	0.0203 (9)	0.0333 (11)	0.0025 (9)
C14	0.0435 (10)	0.0342 (9)	0.0510 (11)	0.0069 (7)	0.0201 (8)	−0.0014 (8)
Cl1A	0.0360 (2)	0.0507 (6)	0.0315 (2)	−0.0031 (4)	0.01848 (17)	−0.0027 (4)
O1A	0.0213 (10)	0.0264 (14)	0.0313 (12)	−0.0038 (8)	0.0111 (8)	−0.0020 (9)
C2A	0.0220 (12)	0.0316 (17)	0.029 (2)	−0.0015 (11)	0.0128 (12)	0.0049 (15)
O2A	0.027 (2)	0.0267 (11)	0.046 (2)	−0.0031 (10)	0.0153 (18)	0.0004 (10)
C3A	0.0236 (13)	0.0302 (15)	0.0333 (14)	−0.0021 (11)	0.0111 (12)	0.0041 (15)
C4A	0.0229 (15)	0.035 (2)	0.0319 (14)	−0.0027 (13)	0.0126 (16)	0.0030 (16)
C5A	0.0271 (18)	0.0292 (11)	0.0313 (13)	0.0024 (13)	0.0155 (14)	0.0023 (9)
C6A	0.0261 (14)	0.0237 (14)	0.0276 (14)	−0.0026 (12)	0.0112 (13)	−0.0005 (11)
C7A	0.0265 (9)	0.0234 (9)	0.0267 (10)	−0.0016 (7)	0.0114 (8)	0.0018 (8)
O7A	0.0312 (17)	0.0333 (12)	0.0271 (16)	−0.0029 (12)	0.0141 (15)	0.0021 (14)
C8A	0.0285 (9)	0.0247 (10)	0.0274 (10)	−0.0002 (7)	0.0137 (8)	0.0014 (7)
C9A	0.0272 (9)	0.0280 (12)	0.0249 (8)	−0.0004 (9)	0.0113 (7)	−0.0016 (9)
C10A	0.0329 (11)	0.0281 (13)	0.0401 (12)	−0.0042 (9)	0.0164 (9)	0.0005 (12)
C11A	0.037 (2)	0.0402 (14)	0.0470 (16)	−0.0115 (12)	0.0215 (17)	−0.0079 (10)
C12A	0.0326 (16)	0.051 (2)	0.045 (2)	−0.0102 (17)	0.0201 (16)	−0.0168 (16)
C13A	0.0251 (14)	0.061 (3)	0.036 (2)	−0.0040 (14)	0.0104 (14)	−0.0124 (16)
C14A	0.0290 (15)	0.0414 (15)	0.0301 (12)	0.0039 (15)	0.0100 (10)	−0.0018 (11)
Cl1B	0.0360 (2)	0.0507 (6)	0.0315 (2)	−0.0031 (4)	0.01848 (17)	−0.0027 (4)
O1B	0.047 (9)	0.023 (7)	0.083 (14)	−0.004 (5)	0.030 (8)	0.000 (6)
C2B	0.055 (11)	0.031 (8)	0.031 (10)	−0.024 (6)	0.021 (9)	−0.024 (7)
O2B	0.017 (9)	0.047 (8)	0.051 (12)	0.008 (5)	0.006 (8)	0.005 (6)
C3B	0.035 (8)	0.042 (9)	0.043 (16)	−0.027 (6)	0.018 (8)	−0.017 (9)
C4B	0.026 (9)	0.019 (7)	0.030 (7)	−0.004 (6)	0.007 (8)	0.002 (5)
C5B	0.020 (9)	0.022 (7)	0.063 (11)	−0.006 (6)	0.019 (8)	−0.004 (6)
C6B	0.029 (7)	0.035 (9)	0.037 (12)	0.007 (6)	0.015 (7)	−0.008 (7)
C7B	0.014 (4)	0.039 (6)	0.020 (5)	−0.001 (4)	0.003 (4)	0.007 (5)
O7B	0.029 (8)	0.023 (5)	0.018 (7)	0.000 (5)	0.010 (7)	−0.001 (6)
C8B	0.032 (5)	0.042 (7)	0.035 (6)	0.001 (5)	0.016 (4)	0.009 (5)
C9B	0.0272 (9)	0.0280 (12)	0.0249 (8)	−0.0004 (9)	0.0113 (7)	−0.0016 (9)
C10B	0.051 (11)	0.024 (7)	0.027 (6)	−0.009 (6)	0.015 (7)	0.005 (5)
C11B	0.033 (13)	0.101 (16)	0.045 (10)	−0.029 (11)	0.020 (10)	−0.019 (9)
C12B	0.021 (8)	0.071 (18)	0.037 (11)	0.011 (8)	0.010 (7)	−0.009 (9)
C13B	0.07 (3)	0.045 (13)	0.044 (14)	0.029 (14)	0.028 (15)	0.019 (10)
C14B	0.046 (15)	0.031 (9)	0.065 (13)	0.000 (8)	0.040 (13)	0.001 (7)

### Geometric parameters (Å, º)

**Table:**

Cl1—C8	1.8162 (15)	O7A—H7AB	0.8400
O1—C2	1.3837 (18)	C8A—H8A	1.0000
O1—C6	1.3668 (18)	C8A—C9A	1.504 (3)
C2—O2	1.2166 (19)	C9A—C10A	1.395 (3)
C2—C3	1.435 (2)	C9A—C14A	1.387 (4)
C3—H3	0.9500	C10A—H10A	0.9500
C3—C4	1.335 (3)	C10A—C11A	1.396 (4)
C4—H4	0.9500	C11A—H11A	0.9500
C4—C5	1.424 (3)	C11A—C12A	1.380 (6)
C5—H5	0.9500	C12A—H12A	0.9500
C5—C6	1.337 (2)	C12A—C13A	1.383 (5)
C6—C7	1.504 (2)	C13A—H13A	0.9500
C7—H7	1.0000	C13A—C14A	1.390 (5)
C7—O7	1.4125 (18)	C14A—H14A	0.9500
C7—C8	1.536 (2)	Cl1B—C8B	1.775 (11)
O7—H7A	0.8400	O1B—C2B	1.399 (18)
C8—H8	1.005 (19)	O1B—C6B	1.356 (16)
C8—C9	1.505 (2)	C2B—O2B	1.235 (18)
C9—C10	1.384 (2)	C2B—C3B	1.417 (17)
C9—C14	1.390 (2)	C3B—H3B	0.9500
C10—H10	0.9500	C3B—C4B	1.310 (17)
C10—C11	1.393 (2)	C4B—H4B	0.9500
C11—H11	0.9500	C4B—C5B	1.395 (14)
C11—C12	1.372 (3)	C5B—H5B	0.9500
C12—H12	0.9500	C5B—C6B	1.314 (14)
C12—C13	1.382 (3)	C6B—C7B	1.512 (15)
C13—H13	0.9500	C7B—H7B	1.0000
C13—C14	1.391 (3)	C7B—O7B	1.458 (17)
C14—H14	0.9500	C7B—C8B	1.478 (12)
Cl1A—C8A	1.823 (2)	O7B—H7BA	0.8400
O1A—C2A	1.388 (4)	C8B—H8B	1.0000
O1A—C6A	1.377 (4)	C8B—C9B	1.504 (12)
C2A—O2A	1.218 (4)	C9B—C10B	1.3900
C2A—C3A	1.443 (4)	C9B—C14B	1.3900
C3A—H3A	0.9500	C10B—H10B	0.9500
C3A—C4A	1.340 (4)	C10B—C11B	1.3900
C4A—H4A	0.9500	C11B—H11B	0.9500
C4A—C5A	1.432 (4)	C11B—C12B	1.3900
C5A—H5A	0.9500	C12B—H12B	0.9500
C5A—C6A	1.342 (4)	C12B—C13B	1.3900
C6A—C7A	1.512 (4)	C13B—H13B	0.9500
C7A—H7AA	1.0000	C13B—C14B	1.3900
C7A—O7A	1.422 (4)	C14B—H14B	0.9500
C7A—C8A	1.531 (3)		
			
C6—O1—C2	121.84 (12)	C7A—C8A—Cl1A	109.13 (12)
O1—C2—C3	116.39 (14)	C7A—C8A—H8A	108.1
O2—C2—O1	116.01 (14)	C9A—C8A—Cl1A	108.45 (14)
O2—C2—C3	127.58 (15)	C9A—C8A—C7A	114.75 (15)
C2—C3—H3	119.6	C9A—C8A—H8A	108.1
C4—C3—C2	120.89 (16)	C10A—C9A—C8A	121.7 (2)
C4—C3—H3	119.6	C14A—C9A—C8A	119.6 (3)
C3—C4—H4	119.8	C14A—C9A—C10A	118.7 (2)
C3—C4—C5	120.40 (16)	C9A—C10A—H10A	119.7
C5—C4—H4	119.8	C9A—C10A—C11A	120.6 (2)
C4—C5—H5	120.5	C11A—C10A—H10A	119.7
C6—C5—C4	118.97 (16)	C10A—C11A—H11A	120.0
C6—C5—H5	120.5	C12A—C11A—C10A	119.9 (3)
O1—C6—C7	111.82 (13)	C12A—C11A—H11A	120.0
C5—C6—O1	121.31 (15)	C11A—C12A—H12A	120.0
C5—C6—C7	126.81 (14)	C11A—C12A—C13A	119.9 (3)
C6—C7—H7	108.2	C13A—C12A—H12A	120.0
C6—C7—C8	111.08 (13)	C12A—C13A—H13A	119.9
O7—C7—C6	111.80 (12)	C12A—C13A—C14A	120.3 (3)
O7—C7—H7	108.2	C14A—C13A—H13A	119.9
O7—C7—C8	109.33 (12)	C9A—C14A—C13A	120.6 (3)
C8—C7—H7	108.2	C9A—C14A—H14A	119.7
C7—O7—H7A	109.5	C13A—C14A—H14A	119.7
Cl1—C8—H8	104.0 (10)	C6B—O1B—C2B	120.5 (18)
C7—C8—Cl1	108.20 (10)	O1B—C2B—C3B	116.5 (19)
C7—C8—H8	110.5 (10)	O2B—C2B—O1B	108 (2)
C9—C8—Cl1	110.65 (10)	O2B—C2B—C3B	130 (2)
C9—C8—C7	113.63 (13)	C2B—C3B—H3B	119.8
C9—C8—H8	109.4 (10)	C4B—C3B—C2B	120 (2)
C10—C9—C8	119.35 (15)	C4B—C3B—H3B	119.8
C10—C9—C14	119.42 (16)	C3B—C4B—H4B	120.6
C14—C9—C8	121.21 (15)	C3B—C4B—C5B	119 (2)
C9—C10—H10	119.9	C5B—C4B—H4B	120.6
C9—C10—C11	120.13 (18)	C4B—C5B—H5B	118.6
C11—C10—H10	119.9	C6B—C5B—C4B	122.8 (17)
C10—C11—H11	119.9	C6B—C5B—H5B	118.6
C12—C11—C10	120.19 (19)	O1B—C6B—C7B	112.2 (15)
C12—C11—H11	119.9	C5B—C6B—O1B	118.7 (16)
C11—C12—H12	119.9	C5B—C6B—C7B	129.1 (16)
C11—C12—C13	120.20 (18)	C6B—C7B—H7B	109.4
C13—C12—H12	119.9	O7B—C7B—C6B	104.2 (15)
C12—C13—H13	120.0	O7B—C7B—H7B	109.4
C12—C13—C14	119.93 (19)	O7B—C7B—C8B	107.1 (14)
C14—C13—H13	120.0	C8B—C7B—C6B	117.1 (12)
C9—C14—C13	120.12 (19)	C8B—C7B—H7B	109.4
C9—C14—H14	119.9	C7B—O7B—H7BA	109.5
C13—C14—H14	119.9	Cl1B—C8B—H8B	108.4
C6A—O1A—C2A	121.9 (3)	C7B—C8B—Cl1B	107.0 (7)
O1A—C2A—C3A	116.2 (3)	C7B—C8B—H8B	108.4
O2A—C2A—O1A	116.4 (4)	C7B—C8B—C9B	113.7 (9)
O2A—C2A—C3A	127.2 (4)	C9B—C8B—Cl1B	110.8 (8)
C2A—C3A—H3A	119.6	C9B—C8B—H8B	108.4
C4A—C3A—C2A	120.7 (4)	C10B—C9B—C8B	122.7 (13)
C4A—C3A—H3A	119.6	C10B—C9B—C14B	120.0
C3A—C4A—H4A	119.5	C14B—C9B—C8B	117.2 (13)
C3A—C4A—C5A	121.1 (4)	C9B—C10B—H10B	120.0
C5A—C4A—H4A	119.5	C9B—C10B—C11B	120.0
C4A—C5A—H5A	120.8	C11B—C10B—H10B	120.0
C6A—C5A—C4A	118.3 (3)	C10B—C11B—H11B	120.0
C6A—C5A—H5A	120.8	C10B—C11B—C12B	120.0
O1A—C6A—C7A	111.4 (3)	C12B—C11B—H11B	120.0
C5A—C6A—O1A	121.5 (3)	C11B—C12B—H12B	120.0
C5A—C6A—C7A	127.1 (3)	C13B—C12B—C11B	120.0
C6A—C7A—H7AA	107.6	C13B—C12B—H12B	120.0
C6A—C7A—C8A	112.6 (2)	C12B—C13B—H13B	120.0
O7A—C7A—C6A	111.7 (3)	C14B—C13B—C12B	120.0
O7A—C7A—H7AA	107.6	C14B—C13B—H13B	120.0
O7A—C7A—C8A	109.7 (2)	C9B—C14B—H14B	120.0
C8A—C7A—H7AA	107.6	C13B—C14B—C9B	120.0
C7A—O7A—H7AB	109.5	C13B—C14B—H14B	120.0
Cl1A—C8A—H8A	108.1		
			
Cl1—C8—C9—C10	−120.90 (15)	C6A—O1A—C2A—C3A	−6.8 (8)
Cl1—C8—C9—C14	60.55 (19)	C6A—C7A—C8A—Cl1A	−54.6 (2)
O1—C2—C3—C4	−4.2 (2)	C6A—C7A—C8A—C9A	−176.5 (2)
O1—C6—C7—O7	−61.88 (17)	C7A—C8A—C9A—C10A	42.4 (3)
O1—C6—C7—C8	60.54 (16)	C7A—C8A—C9A—C14A	−139.4 (2)
C2—O1—C6—C5	−1.8 (2)	O7A—C7A—C8A—Cl1A	−179.6 (3)
C2—O1—C6—C7	−179.31 (12)	O7A—C7A—C8A—C9A	58.5 (3)
C2—C3—C4—C5	0.6 (3)	C8A—C9A—C10A—C11A	177.4 (2)
O2—C2—C3—C4	177.78 (17)	C8A—C9A—C14A—C13A	−176.9 (3)
C3—C4—C5—C6	2.6 (3)	C9A—C10A—C11A—C12A	0.1 (4)
C4—C5—C6—O1	−2.1 (2)	C10A—C9A—C14A—C13A	1.4 (4)
C4—C5—C6—C7	175.07 (16)	C10A—C11A—C12A—C13A	0.1 (4)
C5—C6—C7—O7	120.76 (17)	C11A—C12A—C13A—C14A	0.4 (4)
C5—C6—C7—C8	−116.82 (18)	C12A—C13A—C14A—C9A	−1.2 (4)
C6—O1—C2—O2	−176.92 (13)	C14A—C9A—C10A—C11A	−0.9 (3)
C6—O1—C2—C3	4.8 (2)	Cl1B—C8B—C9B—C10B	−107.5 (10)
C6—C7—C8—Cl1	53.13 (14)	Cl1B—C8B—C9B—C14B	70.4 (11)
C6—C7—C8—C9	176.44 (12)	O1B—C2B—C3B—C4B	−15 (6)
C7—C8—C9—C10	117.14 (16)	O1B—C6B—C7B—O7B	80 (3)
C7—C8—C9—C14	−61.4 (2)	O1B—C6B—C7B—C8B	−162 (2)
O7—C7—C8—Cl1	176.98 (10)	C2B—O1B—C6B—C5B	−11 (5)
O7—C7—C8—C9	−59.71 (16)	C2B—O1B—C6B—C7B	167 (3)
C8—C9—C10—C11	−178.70 (16)	C2B—C3B—C4B—C5B	11 (6)
C8—C9—C14—C13	179.04 (17)	O2B—C2B—C3B—C4B	−167 (5)
C9—C10—C11—C12	−0.3 (3)	C3B—C4B—C5B—C6B	−6 (5)
C10—C9—C14—C13	0.5 (3)	C4B—C5B—C6B—O1B	6 (4)
C10—C11—C12—C13	0.4 (3)	C4B—C5B—C6B—C7B	−172 (3)
C11—C12—C13—C14	0.0 (3)	C5B—C6B—C7B—O7B	−101 (3)
C12—C13—C14—C9	−0.4 (3)	C5B—C6B—C7B—C8B	17 (3)
C14—C9—C10—C11	−0.1 (3)	C6B—O1B—C2B—O2B	173 (4)
Cl1A—C8A—C9A—C10A	−79.9 (2)	C6B—O1B—C2B—C3B	16 (5)
Cl1A—C8A—C9A—C14A	98.3 (2)	C6B—C7B—C8B—Cl1B	61.4 (16)
O1A—C2A—C3A—C4A	3.6 (9)	C6B—C7B—C8B—C9B	−175.9 (15)
O1A—C6A—C7A—O7A	54.5 (5)	C7B—C8B—C9B—C10B	132.0 (10)
O1A—C6A—C7A—C8A	−69.4 (4)	C7B—C8B—C9B—C14B	−50.2 (12)
C2A—O1A—C6A—C5A	5.8 (7)	O7B—C7B—C8B—Cl1B	177.9 (11)
C2A—O1A—C6A—C7A	−173.9 (4)	O7B—C7B—C8B—C9B	−59.5 (15)
C2A—C3A—C4A—C5A	0.7 (10)	C8B—C9B—C10B—C11B	177.8 (12)
O2A—C2A—C3A—C4A	178.7 (7)	C8B—C9B—C14B—C13B	−177.9 (12)
C3A—C4A—C5A—C6A	−2.0 (8)	C9B—C10B—C11B—C12B	0.0
C4A—C5A—C6A—O1A	−1.2 (6)	C10B—C9B—C14B—C13B	0.0
C4A—C5A—C6A—C7A	178.5 (5)	C10B—C11B—C12B—C13B	0.0
C5A—C6A—C7A—O7A	−125.3 (5)	C11B—C12B—C13B—C14B	0.0
C5A—C6A—C7A—C8A	110.8 (4)	C12B—C13B—C14B—C9B	0.0
C6A—O1A—C2A—O2A	177.5 (6)	C14B—C9B—C10B—C11B	0.0

### Hydrogen-bond geometry (Å, º)

**Table:**

*D*—H···*A*	*D*—H	H···*A*	*D*···*A*	*D*—H···*A*
O7—H7*A*···O2*A*	0.84	1.95	2.775 (4)	169
O7—H7*A*···O2*B*	0.84	1.79	2.63 (3)	173
O7*A*—H7*AB*···O2^i^	0.84	2.01	2.835 (6)	170
O7*B*—H7*BA*···O2^i^	0.84	1.86	2.63 (3)	153
C3—H3···O2*A*^ii^	0.95	2.33	3.220 (6)	155
C3—H3···O2*B*^ii^	0.95	2.52	3.42 (4)	158
C3*A*—H3*A*···O2	0.95	2.36	3.236 (5)	153
C5—H5···Cl1^iii^	0.95	2.70	3.6042 (17)	159

Symmetry codes: (i) *x*+1, *y*, *z*; (ii) *x*−1, *y*, *z*; (iii) *x*−1/2, −*y*+1/2, *z*−1/2.
